# Nutritional status of micronutrients as a possible and modifiable risk factor for COVID-19: a UK perspective

**DOI:** 10.1017/S000711452000330X

**Published:** 2020-08-20

**Authors:** David P. Richardson, Julie A. Lovegrove

**Affiliations:** Hugh Sinclair Unit of Human Nutrition, Department of Food and Nutritional Sciences, University of Reading, Whiteknights, Reading RG6 6AP, UK

**Keywords:** COVID-19, Risk factors, Essential micronutrients, Immune functions, Vulnerable groups, 25(OH)D, 25-hydroxyvitamin D, ARTI, acute respiratory tract infection

## Abstract

Recent scientific evidence has indicated that the elderly have increased risk of COVID-19 infections, with over 70s and 80s being hardest hit – especially residents of care homes and in clinical settings, ethnic minorities, people who work indoors and those who are overweight and obese. Other potential risk factors include lack of exposure to sunlight, darker skin pigmentation, co-morbidities, poor diet, certain medications, disadvantaged social and economic status, and lifestyle factors such as smoking and excessive consumption of alcohol. A key question is to understand how and why certain groups of people are more susceptible to COVID-19, whether they have weakened immune systems and what the roles of good nutrition and specific micronutrients are in supporting immune functions. A varied and balanced diet with an abundance of fruits and vegetables and the essential nutrients like vitamin D, vitamin A, B vitamins (folate, vitamin B_6_ and vitamin B_12_), vitamin C and the minerals, Fe, Cu, Se and Zn are all known to contribute to the normal functions of the immune system. Avoidance of deficiencies and identification of suboptimal intakes of these micronutrients in targeted groups of patients and in distinct and highly sensitive populations could help to strengthen the resilience of people to the COVID-19 pandemic. It is important to highlight evidence-based public health messages, to prevent false and misleading claims about the benefits of foods and food supplements and to communicate clearly that the extent of knowledge between micronutrients and COVID-19 infection is still being explored and that no diet will prevent or cure COVID-19 infection. Frequent handwashing and social distancing will be critical to reduce transmission.

The risk of COVID-19 infection and death increases significantly among older people^(1)^, especially those over 70 and 80 years, and those with co-morbidities such as obesity, CVD, hypertension and diabetes^(2)^. Of particular interest is the disproportionate number of deaths in black, Asian and ethnic minorities^(3)^. In the UK, two-thirds of healthcare workers who have died from COVID-19 were from ethnic minorities, including doctors, dentists, nurses, midwives and healthcare support workers^(3)^.

Many of the risk factors identified so far that are related to viral infections and deaths from COVID-19 have underlying associations with nutritional status and specific essential nutrients that are known to contribute to the normal functions of the immune system. Important nutrients that support the immune function include vitamins A and D, the B vitamins (folate, vitamins B_6_ and B_12_) and vitamin C, and the minerals and trace elements Zn, Fe, Se and Cu^(2,4–6)^.

Deficiencies and suboptimal nutritional status of these micronutrients can potentially decrease resistance to infections and reinfections. Deficiencies of nutrients develop progressively over time, and several subclinical stages occur long before clinical symptoms of deficiency appear, including depletion of nutrient stores, biochemical adaptations and impairments of metabolic pathways and functions^(7)^. Validated biomarkers for the micronutrients supporting the immune responses are available and can provide evidence about baseline nutritional status, dietary exposure, impact of nutritional interventions and genetic factors influencing micronutrient metabolism^(8–11)^. Gradually, the different lines of evidence from clinical, nutritional, epidemiological and mechanistic studies are helping to elucidate plausible and biologically relevant relationships between nutritional status and risk of COVID-19.

This evidence can help develop advice for governments and health professionals concerning how best to target vulnerable groups of the population and to identify nutritional policy solutions.

## Vulnerability of people who are deprived of sunlight and are obese

Vitamin D directly affects immune responses, and there are many potential mechanisms by which it may reduce the risks of viral and bacterial infections^(12–15)^. The major source of vitamin D in humans can be via the action of UVB radiation in sunshine on skin, with estimates of cutaneous synthesis providing 80–100 % of the vitamin D requirement of the body with adequate sun exposure^(14)^. Factors that prevent or impede year-round dermal synthesis include season, latitude and prevailing weather conditions, as well as skin pigmentation, age, sunscreen usage, working environment, outdoor activity and sun exposure behaviour. Melanin in skin reduces penetration of UVB and thus contributes to lower vitamin D status in dark-skinned individuals^(14)^. In the absence of sufficient exposure to sunlight, dietary sources of vitamin D, including conventional foods high in vitamin D, fortified foods and food supplements, are necessary to meet nutritional requirements and to avoid vitamin D deficiency. To ensure optimal vitamin D status, use of vitamin D supplements is often required, as sunlight exposure and dietary intake are usually insufficient in many individuals^(15)^. Dermal synthesis of vitamin D may also be less efficient in older people than in younger adults^(13,14,16)^.

People who wear clothes that cover most of their body or who are confined indoors, especially for extended periods of time, and residents in care and nursing homes are very likely to have low exposure to sunlight. In a systematic review of the scientific literature researchers discovered that a high percentage of indoor workers (91 %) had suboptimal vitamin D status, which was highest among shift workers (80 % of individuals) and healthcare students (72 %)^(17)^. Sunlight deprivation accounted for a higher risk of both vitamin D insufficiency and deficiency, with healthcare workers among the most vulnerable groups. The authors concluded that job type may be a major factor in vitamin D deficiency and that guidelines on screening for vitamin D nutritional status and mitigation strategies, such as supplementation, should take into account different jobs and shift patterns. These people, together with those with low intakes of vitamin D in the diet, and those who are socially or economically disadvantaged, are particularly vulnerable to deficiencies and suboptimal nutritional status. Furthermore, naturally rich sources of vitamin D in the diet are few and infrequently consumed. Dietary supply of vitamin D is typically unable to offset the widespread deficiency of UVB-induced synthesis in the skin unless supplemental vitamin D is used^(13,18)^.

There is an association between obesity and low vitamin D status, and obesity and inflammatory conditions increase the risk of vitamin D deficiency^(18)^. In a very recent study, the high prevalence of vitamin D deficiency in people in countries in the Northern Hemisphere exposed to insufficient sunlight has been hypothesised to be linked to a possible role of vitamin D in suppressing the severe inflammatory responses seen in very ill COVID-19 patients and in COVID-19 deaths^(19)^. Obesity is also associated with chronic inflammation in metabolic tissues, and vitamin D is a potent immunomodulator and anti-inflammatory agent. The constant state of low-grade inflammation, characteristic of obesity, may therefore increase requirements for vitamin D^(20)^. The fact that obesity is associated with lower blood 25-hydroxyvitamin D (25(OH)D) concentrations, a recognised biomarker used to assess the risk of vitamin D deficiency and suboptimal intakes^(12–15)^, may be because the lipophilic pool that forms the reservoir of vitamin D in the body is much greater in obese subjects. Such individuals typically need longer exposure to UVB radiation or higher amounts of vitamin D supplementation than those of ideal BMI^(21)^.

## Undernutrition and malnutrition in the community and residents of care and nursing homes

Across the UK, thousands of care homes are experiencing COVID-19 infections and deaths among their residents, many of whom are suffering from dementia. These elderly residents of care homes are acutely vulnerable to COVID-19^(4)^. Undernutrition and malnutrition have been reported over many decades among people in care and nursing homes and in hospital patients^(22–25)^. A significant proportion of hospitalised patients show evidence of impaired nutritional status on admission to hospital including low blood 25(OH)D concentrations and low levels of vitamins B_1_, B_2_, B_6_, folate and vitamin C^(24)^. Patient outcomes are quite likely to be affected by increased medical complications impacting on length of stay and convalescence, severity of the disease, decreased survival rates and ability to live at home^(24)^.

Common nutritional problems in the community and in care homes are low energy intakes, weight loss and vitamin and mineral deficiencies^(26,27)^ due to loss of appetite and age-related loss of senses of taste and smell for reasons including illness, infections and some medications^(28)^. Sarcopenia, depression, co-morbidities such as obesity, changing body composition with increased adiposity and loss of lean body mass, hypertension, diseases of the intestinal tract (resulting in impaired absorption of nutrients), lack of intrinsic factor for absorption of vitamin B_12_, respiratory diseases and cognitive impairments are also common^(24,25)^. Vitamin D deficiency-related co-morbidities also include liver and kidney diseases, and organ transplant recipients are particularly vulnerable^(29,30)^. Psychological and emotional stress such as loneliness, loss of loved ones, anxiety and depression can all have negative effects on physical and mental health and well-being. Medications such as antibiotics, anti-hypertensive, anti-inflammatory, anti-seizure and endocrine drugs are contraindicated for vitamin D and can lead to lower blood 25(OH)D concentrations and interfere with vitamin D functions in the body^(31)^.

## Vitamin D, respiratory and cardiovascular health and optimal nutritional status

The metabolism and actions of vitamin D are well known in relation to bone health, but increasing evidence is emerging about its role in respiratory health, including potential impact on inhibiting pulmonary inflammatory responses and enhancing innate defence mechanisms against respiratory pathogens^(2,5)^. Circulating blood 25(OH)D concentrations have been associated with normal lung function, and there is an increasing evidence-based scientific rationale for the immunomodulant, anti-inflammatory and anti-infective actions of vitamin D^(15)^. For example, in a systematic review and meta-analysis, vitamin D was shown to protect against acute respiratory tract infections (ARTI), and patients who were severely vitamin D deficient experienced the most benefit^(32)^. It has also been shown that there is a linear association between vitamin D status and respiratory infections and lung function^(33)^. The UK Scientific Advisory Committee on Nutrition has recently undertaken a rapid review of vitamin D and ARTI^(34)^. This review noted that currently, the interpretation of the evidence on vitamin D and ARTI was complicated because of differences in vitamin D therapeutic doses and regimens, study settings, participants, study duration, definitions and verification of outcomes (including type of respiratory infection). Since 2017, evidence has suggested that, overall, there is no effect of vitamin D supplementation on reducing ARTI risk, and Scientific Advisory Committee on Nutrition concluded that the evidence at this time does not support recommending vitamin D supplementation to prevent ARTI in the general population. Nevertheless, Scientific Advisory Committee on Nutrition will keep this topic under urgent review if emerging evidence on vitamin D and ARTI risk suggests a change to existing conclusions is warranted^(34)^.

In a review of epidemiological evidence, cardiovascular risks and events have been associated with low vitamin D concentrations^(35)^. Vitamin D is associated with cardiovascular benefits and the regulation of blood pressure with some evidence of its effect on reducing vascular stiffness and vascular dysfunction, although the data are inconsistent^(18)^. Higher vitamin D status has also been associated with a lower risk of type 1 and type 2 diabetes^(18)^. The risk of heart failure has also been reported to be 12-fold higher in elderly people who are vitamin D deficient. This association shows a higher risk than obesity or heart arrythmia^(36)^. Lifestyle factors, such as regular smoking, people living in population-dense urban areas or overcrowded living conditions and in regions impacted by air pollution, people unable to go outside into the sunshine, people who are malnourished, overweight or obese^(37)^ or who drink excessive amounts of alcohol and those with low baseline blood 25(OH)D concentrations, or combinations of all these potentially confounding factors, are likely to have weakened immune systems and functions and be at increased risk of viral infections^(2,4,5,38)^. The benefits of an active outdoor lifestyle and a varied and healthy balanced diet cannot be overemphasised.

## Diet and nutrition surveys and nutritional status of the UK population

The UK National Diet and Nutrition Surveys of nutrient intake and nutritional status published in 2019^(39)^ have shown a sustained worsening of the dietary intakes and chronic shortages of several of the nutrients involved in supporting the normal immune functions, including vitamin D, vitamin B_12_, Zn, Se, Cu and vitamins A and C. Low intakes of these essential nutrients over a 9-year period are evident in the general population of all age/sex groups. For example, an analysis of UK Biobank data^(40)^ found that vitamin D intakes from the diet of Bangladeshi, Indian and Pakistani individuals ranged from 1·0 to 3·0 µg/d – well below the Public Health England and Scientific Advisory Committee on Nutrition recommendations for 10 µg/d^(16)^ and the European Food Safety Authority adequate intake of 15 µg/d^(41)^. Vitamin D supplementation was low amongst UK South Asians, with only 22 % of Bangladeshis, 32 % of Indians and 25 % of Pakistanis taking a vitamin D supplement. Within this group of South Asians, women (39 %) were more likely to take a supplement than men (23 %)^(40)^. Interestingly, an analysis of the UK Biobank cohort showed a very high prevalence of low levels of blood 25(OH)D and vitamin D deficiency in UK/South Asian adults^(37)^. However, in a recent study, the UK Biobank Data provided no evidence that plasma 25(OH)D concentrations explained susceptibility to COVID-19 infection, either overall or observed differences between ethnic groups^(42)^. This conclusion has been subsequently challenged^(43)^, and further clinical and epidemiological studies are required to unravel the conflicting results.

The National Diet and Nutrition Surveys findings in 2018 of the micronutrient intakes for adults aged 65–70 years and 75 years and over showed that for vitamin D, mean intakes were 35 % of the Reference Nutrient Intake for adults aged 65–74 years and 28 % Reference Nutrient Intake for those over 75 years. Inclusion of intakes from vitamin D food supplements brought the mean intakes up to 60 and 53 % for those age groups, respectively^(44)^.

Vegetarian and vegan diets^(45)^ can also generally be low in nutrients such as vitamin B_12_, Zn, Cu, Fe and Se as well as vitamin D and the *n*-3 long-chain fatty acids DHA and EPA found mainly in oily fish and cod liver oil. In fact, the high prevalence of vegetarianism and the avoidance of all foods of animal origin in Indian and ethnic minorities could exacerbate deficiencies and suboptimal intakes of all these essential nutrients. For example, Zn bioavailability is influenced by high-fibre diets and their phytate content because of the binding of Zn by these components in plant-based foods, particularly grains and legumes. Dietary phytate is known to be a contributory factor for Zn deficiency^(46,47)^.

In a study of seven Western countries of intakes and deficiencies of eight trace elements in older adults (≥60 years)^(27)^, Se deficiency was observed in 49 % of women and 37 % of men living in the community and 44 % of women and 27 % of men in care homes, nursing homes and retirement homes. Zn deficiency was also observed in 31 % of community-based women and 49 % of men. Significant proportions of both older populations showed insufficiencies not only of Zn and Se but also for Fe, iodine and Cu^(27)^.

For Se, the 2018 National Diet and Nutrition survey^(44)^ showed that a substantial proportion of older adults in all age/sex groups had intakes below the lower nutrient reference intake, that is high risk of deficiency. Of those aged 75 years or older, 34 % of men and 57 % of women had intakes below the lower nutrient reference intake. Of women aged 75 years and over, 12 % had intakes below the lower nutrient reference intake for Fe and Zn compared with 8 and 3 %, respectively, in women aged 65–74 years.

## Nutrients contributing to the normal functions of the immune system

Several essential nutrients contribute to the normal functions of the immune system. Zn has a role in antiviral immunity^(48)^, and deficiency of dietary Zn has been reported to increase susceptibility to infectious diseases. Zn has also been given as an effective adjunct treatment in reducing mortality from severe pneumonia^(49)^.

The risk of viral infections and several chronic diseases is modified by low Se intakes and nutritional status^(50,51)^. Selenoproteins such as glutathione peroxidase play a crucial role in the body’s response to oxidative stress^(50,51)^. During viral infection, the pathogens induce oxidative stress by generating reactive oxygen species in host cells, which, if not counterbalanced by antioxidant defence mechanisms, leads to oxidative stress and the emergence of more virulent strains^(48,52)^. The Se content of plant and animal products can vary 10-fold according to soil and geographic origin, and the lowest Se status is found in populations that eat vegetarian diets including plants grown in low-Se areas of the world^(53)^. Potentially relevant to the recent appearance of COVID-19 in China is the association between lower regional Se nutritional status and more severe reported outcomes of COVID-19 cases in different regions of China^(53)^, although that does not prove cause and effect. Multiple cellular and viral mechanisms involving Se and selenoproteins could influence viral pathogenicity, severity and duration of respiratory symptoms, recovery and death rates from COVID-19^(52)^.

Cu-related enzyme cytochrome c oxidase is needed for energy production of immune cells and another cupro-enzyme, superoxide dismutase, plays a role in the protection of immune cells against reactive oxygen species. Moderate or even marginal Cu deficiency affects some activities of T lymphocytes and phagocytic cells adversely^(54,55)^. Although evidence for impaired function of the immune system resultant from Fe deficiency in humans is scarce, changes have been demonstrated in *in vitro* tests and animal models. These mechanistic observations resulted in European Food Safety Authority concluding that a cause and effect association has been established between dietary status of Fe and a normal immune function^(56)^.

In addition to the functions of vitamin D, vitamin A has crucial immunomodulatory roles, and vitamin A metabolites exert direct effects on immune functions^(47,57)^. Vitamin A deficiency is associated with impaired intestinal immune responses and increased risk of infectious morbidity and mortality in relation to gastrointestinal and respiratory infections. Preformed vitamin A (retinol) is only found in animal-derived foods. Carotenoid precursors of vitamin A, such as β-carotene, are abundant in green leafy vegetables and certain fruits, but intakes of fruits and vegetables in the UK are still well below the recommended number of five-a-day servings. The National Diet and Nutrition Surveys 2019 states that the proportion of people meeting the five-a-day target remains low, at about 30 % for adults and about 10 % for 11- to 18-year-olds^(39)^.

There is no evidence of direct immune impairments in vitamin E-deficient individuals and a restoration of a depressed immune system by the vitamin^(58)^, although vitamin E has important immunomodulatory effects and may confer protection against infectious diseases^(59)^. For example, clinical outcomes demonstrate a role for vitamin E in reducing the risk of respiratory tract infections by improving immune response in elderly nursing home residents^(60,61)^.

Vitamin C contributes to normal immune functions and the inhibition of inflammatory processes^(62)^. Vitamin C deficiency results in impaired immunity and higher susceptibility to infections. In turn, infections significantly impact on vitamin C levels due to enhanced inflammation and increased metabolic requirements^(62)^. Vitamin C appears to exert a number of beneficial effects on cellular functions of both the innate and adaptive immune systems^(62)^. In addition, it has a very effective antioxidant role *per se*, but it is also needed for the regeneration of vitamin E^(62)^. There are numerous factors that can affect vitamin C status, including dietary intake, obesity, severe infections, institutionalisation and smoking^(63)^. Smokers with low intakes of vitamin C show increased oxidative stress, and plasma vitamin C is decreased by about 40 % in male smokers^(62)^. Vitamin C status, as measured by plasma and leucocyte concentrations, is lower in elderly people with chronic illnesses, especially in care and medical settings, compared with young adults^(62)^.

Other important water-soluble vitamins that contribute to the normal immune functions are the B vitamins folate, B_6_ and B_12_^(64–66)^. Vitamin B_6_ is required as a coenzyme in the metabolism of antibodies and cytokines. Lymphocytes isolated from vitamin B_6_-deficient people display reduced proliferation, reduced IL-2 production in response to mitogens and reduced antibody production in response to immunisation^(65,67)^.

Folate plays a crucial role in nucleotide synthesis and thus may affect immune cell proliferation and responsiveness. Folate deficiency has been shown to reduce proliferation of various cell types and to reduce the proportion of circulating T lymphocytes and their proliferation in response to mitogen activation. *In vitro* studies demonstrated that all the effects on the immune system were reversible either by folate addition or nucleotide repletion^(64,68)^.

Vitamin B_12_, along with vitamin B_6_ and folate, is involved in the immune functions through their involvement in nucleic acid and protein biosynthesis. Decreased availability of vitamin B_12_ for rapidly proliferating B lymphocytes is believed to result in an impaired antibody response to pneumococcal polysaccharide vaccine and synthesis of specific Ig^(66,67)^.

Based on scientific assessments carried out by European Food Safety Authority, the European Register of authorised nutrient function health claims^1^ ^(69)^ include the specific nutrients vitamin A (including β-carotene), vitamin D, the B vitamins (folate, vitamins B_6_, B_12_), vitamin C and the essential minerals and trace elements Fe, Cu, Zn and Se for their contributions to the normal functioning of the immune system^(69)^. However, to date, there have been no direct associations between status of these micronutrients and COVID-19 infection.

## Strategies to avoid nutritional deficiencies and ensure adequacy of micronutrient status in different populations

The primary public health and nutrition strategy is to provide advice on the importance of a varied and balanced diet and a healthy lifestyle. However, when there is evidence of non-compliance with this advice, and where diet and nutrition surveys demonstrate inadequate micronutrient intakes and prevalence of low nutritional status, national nutrition strategies also include the addition of micronutrients to commonly eaten foods such as vitamin D added to margarines and fat spreads (food fortification) and the use of food supplements^(13,70)^. Overall, micronutrient status, particularly vitamin D status, may be exacerbated during this COVID-19 pandemic as a result of the restrictions on movements, which may impair normal immune functions^(2,4–6,71)^. Very recently, studies have shown that vitamin D plays a role in regulating and suppressing the cytokine inflammatory response that causes the acute respiratory distress syndrome observed in severe and often lethal forms of COVID-19^(71)^. In addition, a significant correlation has been shown between low serum vitamin D levels and mortality from COVID-19^(72,73)^.

As this review illustrates, vitamin D is not the only essential nutrient known to contribute to normal functioning of the immune system^(2,5,74)^. It has been suggested by some researchers that, in addition to the consumption of a well-balanced diet, other nutrients such as vitamin C, Zn and the *n*-3 fatty acids EPA + DHA at levels above the daily reference values, may help reduce nutritional gaps, support optimal immune functions and possibly reduce risk and consequences of infection^(2,5)^. Dietary approaches to achieve a healthy gut microbiota may also benefit the immune system^(71)^.

In recent reviews of the therapeutic uses of vitamin D to prevent or treat ARTI, and data from human intervention trials on COVID-19 patients, using pharmacological doses, there was no evidence of any specific therapeutic effect. However, both reports^(34,75)^ advised that all people should continue to follow the UK Government advice on daily vitamin D supplementation of 10 µg/d to maintain bone and muscle health during the COVID-19 pandemic, since people may not be getting enough from sunlight exposure or diet. In a further rapid review, the Royal Society^(76)^ agreed that there is no direct causal link yet between vitamin D deficiency and increased susceptibility to COVID-19. However, this report pointed out that the UK has one of the highest levels of vitamin D deficiency in Europe and that it would be prudent for the Government to provide a stronger public health message about avoiding vitamin D deficiency. Despite social media attention and claims for high-dose therapeutic benefits of some nutrients, it is key to prevent false or misleading claims^2^ ^(5,6)^ that diet or individual micronutrients can either ‘boost’ the immune system or ‘prevent’ or ‘cure’ COVID-19 infection. Indeed, medicinal claims for prevention, cure and alleviation of a disease for foods and food supplements are illegal in the UK and the European Union as a whole^(77)^. Furthermore, consumption of excessive quantities of some micronutrients can have adverse metabolic and health effects, and total intakes of each nutrient from all food and food supplement sources must take into account the tolerable upper safe level set by expert scientific committees such as European Food Safety Authority^(78)^, the Food and Nutrition Board/Institute of Medicine in the USA^(31,47,79)^ and the UK Expert Vitamin and Mineral Group^(80)^.

In conclusion, avoidance of nutrient deficiencies, identification of target groups at high risk of suboptimal nutritional status and the use of practical, safe and effective nutrition policy solutions may help strengthen the resilience of people to the COVID-19 pandemic. Further research and the greater understanding of potential nutritional risk factors could help explain why certain groups of people are more susceptible to COVID-19. The evolving information will contribute to the evidence base for dietary counselling on healthy weight and the role of the essential micronutrients in supporting immune functions, as well as for the development of clinical guidelines and effective public health strategies on nutrition and health.

## Footnotes

1 A form of claim that refers to the physiological role of the nutrient in growth, development and normal functions of the body (Regulation EC 1924/2006).

2 Claim means any message or representation, including pictorial, graphic or symbolic representation, in any form which states, suggests or implies that a food has particular characteristics (Regulation EC 1924/2006).
